# The effects of a single bout of high intensity exercise on stress reactivity, mind wandering, and lecture comprehension in young adults

**DOI:** 10.1371/journal.pone.0318222

**Published:** 2025-01-31

**Authors:** Anisa Morava, Ali Shirzad, James Van Riesen, Mustafa Shirzad, Nader Elshawish, Erind Alushaj, Harry Prapavessis

**Affiliations:** 1 School of Kinesiology, Western University, London, Ontario, Canada; 2 Department of Neuroscience, Schulich School of Medicine and Dentistry, Western University, London, Ontario, Canada; Universidad de Concepción Facultad de Medicina: Universidad de Concepcion Facultad de Medicina, CHILE

## Abstract

Post-secondary students experience acute stressors daily. Acute stress has been associated with poor cognitive and learning outcomes. Prior work has demonstrated a single bout of exercise can attenuate acute stress responses. The present study examined the effects of a single 30-minute bout of high intensity aerobic exercise on multidimensional stress reactivity and learning-related outcomes. Forty participants were randomized to either engaging in an exercise bout or seated rest. Participants were then exposed to the Trier Social Stress Test followed by a 20-minute video lecture. The video lecture contained embedded mind wandering probes. Acute exercise did not attenuate stress responses, however promoted greater on-task behaviour (i.e., less mind wandering) and improved lecture comprehension scores. Notably, state anxiety was positively associated with mind wandering and mind wandering was negatively associated with lecture comprehension. Collectively, examining the role of acute interventions that reduce state anxiety may promote favourable learning outcomes in young adults.

## Introduction

Post-secondary students frequently experience a variety of stressors and report high stress levels in relation to the general population [1–3]. Acute and chronic stressors have been well-documented to negatively impact several life domains including health (i.e., physical, mental), relationships, work, and education [4,5]. In relation to education, acute stressors have been associated with poor cognitive and academic outcomes [6,7]. Importantly, several experimental studies have highlighted that acute stress can be debilitative or facilitative to cognitive function depending on acute stressor intensity, the temporal relation between the acute stressor and the cognitive assessment(s), the specific cognitive domain, and individual factors [8].

Evidence for the debilitative effects of acute stress has been documented for several cognitive domains including attention, memory, and learning [8–10]. For example, when acute stress was induced in young male adults via the socially evaluated cold pressor test (CPT) *prior* to a cognitively demanding luminance and orientation detection task, individuals exhibited impaired attentional allocation and “strong distractibility” [11]. Meta-analytic evidence from Shields and colleagues (2017) suggested that when the acute stressor occurred *prior to or during encoding* it impaired episodic memory (i.e., “summary records of experience”) [9,12] unless the stressor-encoding delay was brief or in instances where the content of the study materials was related to the stressor. When considering acute stressors in a post-secondary learning context, previous work from our lab suggests that young adults exhibit greater mind wandering (i.e., task unrelated thoughts) and reduced lecture comprehension if acute stress is induced via Trier Social Stress Test (TSST) *prior* a video lecture [13]. As success in the post-secondary learning context is often predicated on attention during lectures and subsequent lecture comprehension [14], identifying practical and effective interventions to manage acute stress responses is critical for student well-being and success.

Physical activity (PA) and exercise may be one such intervention to manage stress responses. Several systematic reviews and meta-analyses [15–18] provide evidence that both regular and a single bout of exercise can reduce reactivity to acute stressors. The meta-analyses by Hamer et al. (2006) and Mariano et al. (2022) provide evidence that a single bout of exercise buffers systolic and diastolic blood pressure elevations in response to acute stress exposure, albeit to a small degree. Importantly, encountering a stressor activates several physiological and psychological systems including the Hypothalamic Pituitary Adrenal (HPA) and Sympathetic Adrenal Medullary (SAM) axes [19] warranting the assessment of stress reactivity using a multidimensional lens [18,20]. In a systematic review by Morava and colleagues (2024), acute exercise yielded the most robust reductions to blood pressure and cortisol responses to acute stressors, with mixed evidence for heart rate and negligible effects on self-reported stress [18]. Prior studies examining the role of exercise intensity and duration on stress reactivity have suggested moderate to high intensities for durations of approximately 30 minutes yield the greatest reductions specifically to blood pressure [16] and salivary cortisol measures [21].

Given the evidence of negative effects of acute stress on learning processes and the stress buffering effects of a single bout of exercise, the present study examined the effects of a 30-min, high intensity aerobic exercise bout on stress reactivity and the downstream effects on mind wandering and lecture comprehension in young adults. We predicted that individuals who engaged in exercise prior to the stressor would exhibit reduced state anxiety, blood pressure and cortisol reactivity, reduced mind wandering, and improved lecture comprehension.

## Method

### Participants

As there were several variables of interest (i.e., mind wandering, lecture comprehension) an *a priori* sample size of 40 participants was generated from the smallest reported effect size comparable to lecture comprehension (i.e., cognitive performance) of ηp^2^ = 0.05 [22], powered at 0.80, with an alpha of 0.05 using G*Power 3 software [23]. The sample size generated was based on the reported effect size on cognitive performance as this is a critical outcome in the context of post-secondary learning environments. Participants were recruited from Western University, Canada via mass emailer, which is sent to all undergraduate and graduate students at the institution and online advertisements. Exclusion criteria were as follows: >30 years of age, use of tobacco, marijuana, or other substances [24,25] use of prescription medications for chronic health conditions (e.g., cortisol-related disorders [26,27] use of prescription medications for depression or anxiety [28] pregnancy and/or currently breastfeeding, diagnosis of a learning-related condition (i.e., ADHD, dyslexia), inability to engage in thirty minutes of physical activity (as screened by the Physical Activity Readiness Questionnaire for Everyone – PARQ+), illness (e.g., fever) on the day of the study [29], and prior viewing of the presented video lecture. Several of the above exclusion criteria are associated with salivary cortisol. Specifically, tobacco and marijuana use have been associated with increased salivary cortisol [24,25]. Alcohol use has been associated with changes to salivary cortisol concentrations and the cortisol awakening response [25]. Depression and anxiety have been associated with HPA axis dysregulation, such as hypercortisolemia [27,28]. Cortisol-related disorders, such as Cushing’s syndrome, are characterized by overproduction of cortisol by the adrenal glands [27] and illness on day of testing has been demonstrated to impact cortisol dynamics [29]. Diagnosis of a learning-related condition and prior viewing of the presented video lecture were further exclusion criteria due to the potential impact of the above on mind wandering and lecture comprehension.

See Table 1 for participant demographics.

**Table 1:** Demographics.

	*Control*	*Exercise*
N	20	20
Age	20.65 (2.72)	21.4 (2.93)
Sex (no. females)	11	11
Years of Education	14.8 (2.04)	15.15 (2.03)
GPA	3.69 (0.23)	3.56 (0.41)
Prior Lecture Attendance (no.)	6	5
Sleep Duration (h)	7.15 (1.30)	7.89 (1.33)
OC Use (no.)	5	3
Menstrual Phase (no. in follicular phase)	5	4
GLTEQ Score	59.95 (24.44)	56.45 (29.99)
PSS	14.05 (6.14)	17.4 (6.39)
STAI-T	36.05 (9.51)	41.14 (10.2)

Note. Values reflect means and standard deviations in parentheses. no.- number of, OC use - Oral contraceptive use, GLTEQ - Godin Leisure Time Exercise Questionnaire, PSS - Perceived Stress Scale, STAI-T - State Trait Anxiety Inventory – Trait. Twenty two participants identified as women and 18 participants identified as men.

Participants provided informed written consent of a protocol approved by the Health Sciences Research Ethics Board (#122279) at Western University. This study was conducted in accordance with the most recent iteration of the Declaration of Helsinki. The recruitment and data collection for this study was conducted between February 13^th^, 2023, and March 23^rd^, 2023.

### Materials

#### Exercise intervention.

The exercise intervention consisted of 30 minutes of aerobic treadmill exercise at an intensity of 70% of participant heart rate reserve (HRR) based on a procedure outlined by Caplin and colleagues (2021) [21]. Participant’s HRR was calculated according to the following formula: HRR = Age-Predicted Heart Rate (HR) Maximum - Resting Heart Rate, where Age-Predicted HR Maximum = 220 – age [30]. Resting Heart Rate was taken during the baseline assessments and once again before the exercise began. As HR often fluctuates, participants were expected to stay within 10 percent of 70% HRR. A researcher supervised the exercise session and adjusted the speed and incline of the treadmill to ensure participants remained within this range. Participants engaged in a 2.5 min warm-up walk, 25-min at 70% HRR, and 2.5 min cool-down walk.

#### Acute stress induction.

The Trier Social Stress Test (TSST) has been used in numerous studies to induce an acute stress response [31,32]. Participants were informed that they would need to prepare and present a speech describing why they would be an ideal candidate for their ideal job to a panel of two judges trained in public speaking. A camera was visible during the speech to induce a state of social evaluation; however, the camera did not record participants’ performance. The participant was then left alone for 10-min to prepare their speech before the judging panel returned. Participants then delivered their 5-min speech after which they were instructed to perform an arithmetic task consisting of serial subtractions of thirteen from 1022 (i.e., 1022 minus 13; 1009 minus 13; etc.,) in front of the same judging panel. The serial subtractions lasted for 5-min, see [33] for in-depth details on the TSST protocol.

#### Lecture.

Participants viewed a twenty-minute video lecture presented by a Nobel laureate. Video lectures of a comparable duration (i.e., twenty minutes) have been used in prior mind wandering studies [13,34]. The video lecture covered the economics of climate change and was selected from a publicly available YouTube channel. Participants were not permitted to take notes during the lecture.

### Measures

#### Demographics.

Age, sex, gender, education (i.e., years of education), grade point average (GPA), prior lecture attendance the day of the study, hours of sleep the night prior to the study, oral contraceptive use, and menstrual cycle phase (i.e., follicular, luteal) were collected. The Godin Leisure Time Exercise Questionnaire (GLTEQ) [35] was used as a measure of weekly self-reported exercise. Greater GLTEQ values indicate greater exercise engagement. The Perceived Stress Scale (PSS) [36] was used to assess perceived stress in the past month. The PSS includes ten items assessing the degree to which experiences in one’s life are considered stressful. Greater PSS scores indicate greater levels of perceived stress. The calculated Cronbach’s α for the PSS was α = 0.880.

#### State and trait anxiety.

The State Trait Anxiety Inventory (STAI) [37] was used to measure state and trait anxiety. The STAI-S consists of twenty items measuring state anxiety and the STAI-T consists of twenty items measuring trait anxiety. The calculated Cronbach’s α for STAI-S and STAI-T were α = 0.877 and α = 0.877 respectively.

#### Heart rate.

Heart rate was assessed via a heart rate monitor (Polar H10 Wearlink + Coded Transmitter, Polar Electro Inc., Lake Success, NY, USA).

#### Blood pressure.

Blood pressure was assessed via an electronic blood pressure cuff (Omron BP7455CAN, Omron Healthcare Co., Kyoto, Japan).

#### Salivary cortisol.

Participants provided saliva samples ( ∼ 0.5 mL) using a passive drool method. Immediately after collection, the saliva vials were stored in a – 80 °C freezer until assayed in duplicate using a high sensitivity enzyme immunoassay (Salimetrics LLC, Carlsbad, CA) according to manufacturer instructions. The intra-assay CV was 4.6% and the inter-assay CV was 6.00%. Sensitivity for these assays was 0.007 µg/dL. Cortisol concentrations were converted from µg/dL to nmol/L to be in line with the human salivary cortisol literature.

#### Mind wandering.

Probe-caught mind wandering was assessed via a five-point Likert scale used in prior mind wandering studies see [38,39]. The five points on the scale are described as follows: 1 = Completely on task, 2 = Mostly on task, 3 = Both on task and on unrelated concerns, 4 = Mostly on unrelated concerns, and 5 = Completely on unrelated concerns [38]. Three mind wandering probes were distributed equally throughout the lecture as conducted in prior work [13,40]. The screen provided the following instruction: “Which of the following responses best characterizes your mental state just before this screen appeared?”. At the end of the video lecture participants also reported how many minutes they perceived they were mind wandering if at all and to what extent they thought about the stressor during the lecture on a scale of 1 = Not at all to 5 = A great deal.

#### Lecture comprehension.

Lecture comprehension was assessed via 20 purpose-built multiple-choice questions.

### Procedure

**Fig 1:**
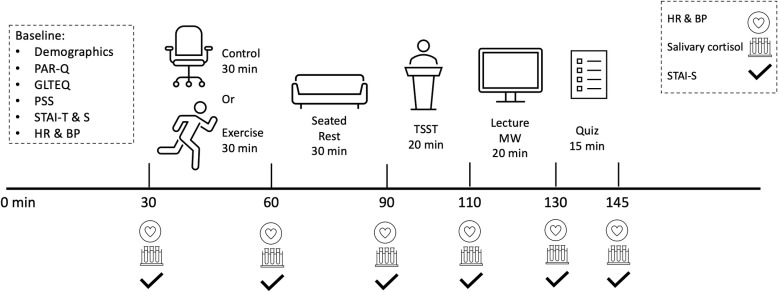
Study schematic.

Participants abstained from exercise and caffeine 3 h prior to lab arrival and food or beverage consumption (except water) for 1 h prior to lab arrival [41]. Participants also abstained from dental work the day prior and brushing teeth for 1 h prior to lab arrival. All study procedures were completed between 12:00 to 6:00 pm to minimize circadian variation in salivary cortisol [42]. Participants were randomly assigned via simple randomization to a Control (n = 20) or Exercise (n = 20) condition using the online randomizer tool (random.org). Evaluators were unaware of group assignment. Fig 1 represents a schematic of the study events.

Upon entry into the lab, participants were seated and filled out the demographic questionnaire, PAR-Q, GLTEQ, PSS, STAI-T, and STAI-S. BP was assessed twice in a seated position and participants were fitted with a heart rate monitor to provide continuous HR measurement. Participants were instructed on how to passively drool and provided their first saliva sample. These baseline assessments took approximately 30 minutes, which promoted acclimation to the lab environment prior to the Control or Exercise condition [43]. HR, BP, state anxiety, and a saliva sample were collected again prior to either condition. Participants in the Control condition remained in a seated position for 30 minutes in the lab with the experimenter and were not permitted to use technology (i.e., smartphones or laptops) during this time. Participants in the Exercise condition engaged in 30 minutes of aerobic treadmill exercise with the experimenter adjusting the treadmill speed/incline to ensure the participant’s heart rate remained within the 70% HRR target. Following either condition, HR, BP, state anxiety, and another saliva sample were collected. Following the above assessments, participants remained in a seated position for 30 minutes in the lab with the experimenter and once again were not permitted to use technology during this time. Following this 30-min rest period, participants’ HR, BP, state anxiety, and saliva sample were collected.

All participants were then exposed to the TSST in a separate room in the lab. Following the TSST, the above stress measures were repeated. Participants were then instructed that the next task involved watching a twenty-minute video lecture in which three prompts about their mental state (i.e., mind wandering probes) would appear. Participants were also informed they were not permitted to take notes and that a lecture comprehension assessment would follow. Immediately following the lecture, HR, BP, state anxiety, and a saliva sample were collected. Participants then provided self-reported mind wandering minutes and rumination during the video lecture. Participants were then presented with a paper and pencil lecture comprehension assessment and instructed they had 15 minutes to complete the assessment. Each correct answer on the paper and pencil assessment was awarded one point out of twenty possible points. Immediately following the lecture comprehension assessment, HR, BP, state anxiety, and a final saliva sample were collected. Participants were debriefed regarding the true purpose of the study and compensated via a $20 CAD Amazon gift card. Materials are available by emailing the corresponding author while code for the lecture video with embedded mind wandering prompts is openly available on GitHub (https://github.com/Morava83/DataCollection).

### Results

#### Statistical analyses.

All variables were assessed for normality. Greenhouse-Geisser corrections for violations of sphericity are reported where appropriate (corrected degrees of freedom reported to one decimal place) and an alpha level of 0.05 was used for all ANOVA models. Effect sizes (i.e., Cohen’s *d*, Partial eta-squared, *r*) are reported where appropriate. Boxplots for dependent variables were constructed and inspected to screen for potential outliers to winsorize (Q3 + 1.5* Interquartile range or Q1 – 1.5* Interquartile range). All analyses were conducted using SPSS Version 29. HR, BP, and STAI-S values were assessed via separate repeated mixed-model ANOVAs with a between-subject factor of Group (i.e., Control, Exercise) and a within-subject factor of time. As salivary cortisol data was non-normally distributed, a log-transformation was applied to reduce skewness. Overall cortisol secretion was calculated via the “area under curve with respect to ground” formula (AUCg) (44) from the post-Sit sample. Area under the curve with respect to increase (AUCi) was derived from the AUCg values as outlined by Pruessner [44]. Cortisol reactivity was calculated as the difference between the Post-Sit sample and the participant’s cortisol peak (i.e., maximum) sample [45]. Cortisol metrics were assessed using independent t-tests between the Control and Exercise groups. As probe-caught mind wandering and rumination scores were non-normally distributed, Mann-Whitney U tests were used. Independent t-tests were used to examine mind wandering minutes and lecture comprehension scores. Pearson and Spearman correlations were conducted to assess the strength of the associations between key variables (e.g., stress responses, mind wandering, and learning).

#### Group equivalency.

There were no reliable differences between participants assigned to the Stress versus Exercise group with respect to age, sex, education, GPA, sleep the night before, prior lecture attendance, oral contraceptive use, menstrual phase, GLTEQ score, PSS score, STAI-T score, and baseline physiological measures (i.e., HR, SBP, DBP, pre-TSST cortisol), all *p*’s > 0.05.

### Stress response analyses

#### HR.

**Fig 2:**
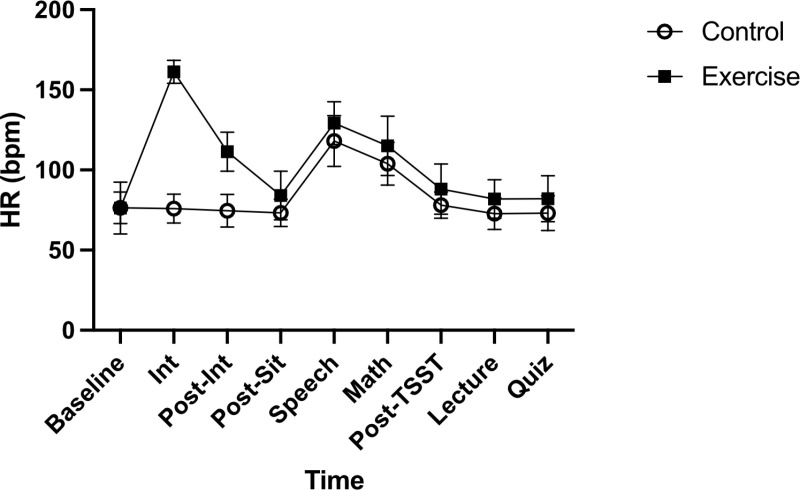
Heart rate (HR) values in beats per minute (bpm) are depicted for the Control and Exercise group. Error bars represent ± SD. * p < 0.05 (between groups).

There was a significant Time by Group interaction for HR, *F*(5.0, 192) = 118, *p* < 0.001, ηp^2^ = 0.76. Results yielded a significant main effect of Group on HR, *F*(1,38) = 38.1, *p* < 0.001, ηp^2^ = 0.50. There was also a significant main effect of Time for HR, *F*(5.0,192) = 250, *p* < 0.001, ηp^2^ = 0.87. The Exercise group had significantly higher HR than the Control group only at the Intervention, *t*(38) = 21.3, *p* <.001, and post-interven*t*ion, *t*(38) = 9.2, *p* < 0.001, timepoints following Bonferroni correction (Fig 2).

#### SBP.

There was a significant Time by Group interaction for SBP, *F*(5, 190) = 2.98, *p* = 0.013, ηp^2^ = 0.07. Results yielded no significant main effect of Group for SBP, *F*(1,38) < 1, but a significant main effect of Time for SBP, *F*(5,190) = 14.2, *p* < 0.001, ηp^2^ = 0.27. The Exercise group had no significant differences in SBP than the Control group at each of the time points (all *t*-sta*t*istics were less than 2.1 and *p-*values > 0.05).

#### DBP.

There was no significant Time by Group interaction for DBP, F(3.5,132) = 2.29, *p* = 0.07, ηp^2^ = 0.06. Results yielded no significant main effect of Group for DBP, *F*(1,38) = 2.48, *p* = 0.12, ηp^2^ = 0.06. Results yielded a significant main effect of Time for DBP, F(3.5,132) = 4.12, *p* = 0.005, ηp^2^ = 0.098.

#### STAI-S.

**Fig 3:**
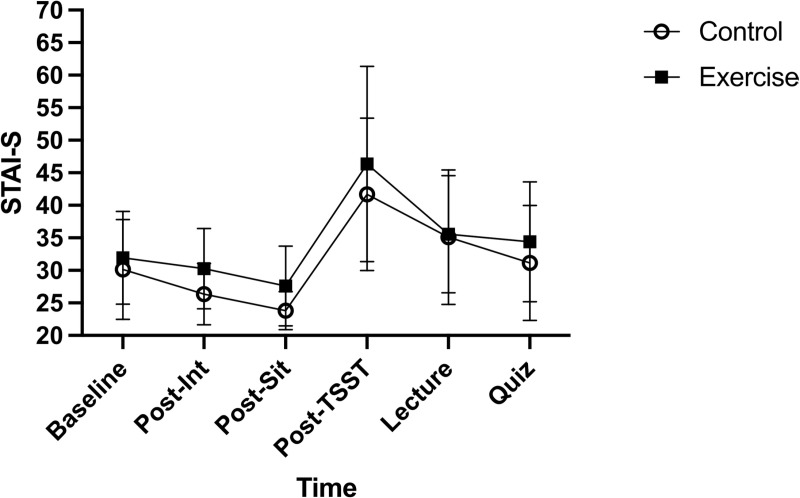
State anxiety (STAI-S) values are depicted for the Control and Exercise group. Error bars represent ± SD.

There was no significant Time by Group interaction for STAI-S, *F*(2.2,84.8) < 1, ηp^2^ = 0.02. Results yielded no significant main effect of Group for STAI-S, *F*(1,38) = 2.19, *p* = 0.15, ηp^2^ = 0.06. Results yielded a significant main effect of Time for STAI-S, *F*(2.2,84.8) = 37, *p* < 0.001, ηp^2^ = 0.49. See Fig 3.

#### Cortisol.

Two participants displayed physiologically implausible values of cortisol and were removed from cortisol-based analyses only.

#### Overall cortisol output (AUCg), (AUCi), and cortisol reactivity.

**Fig 4:**
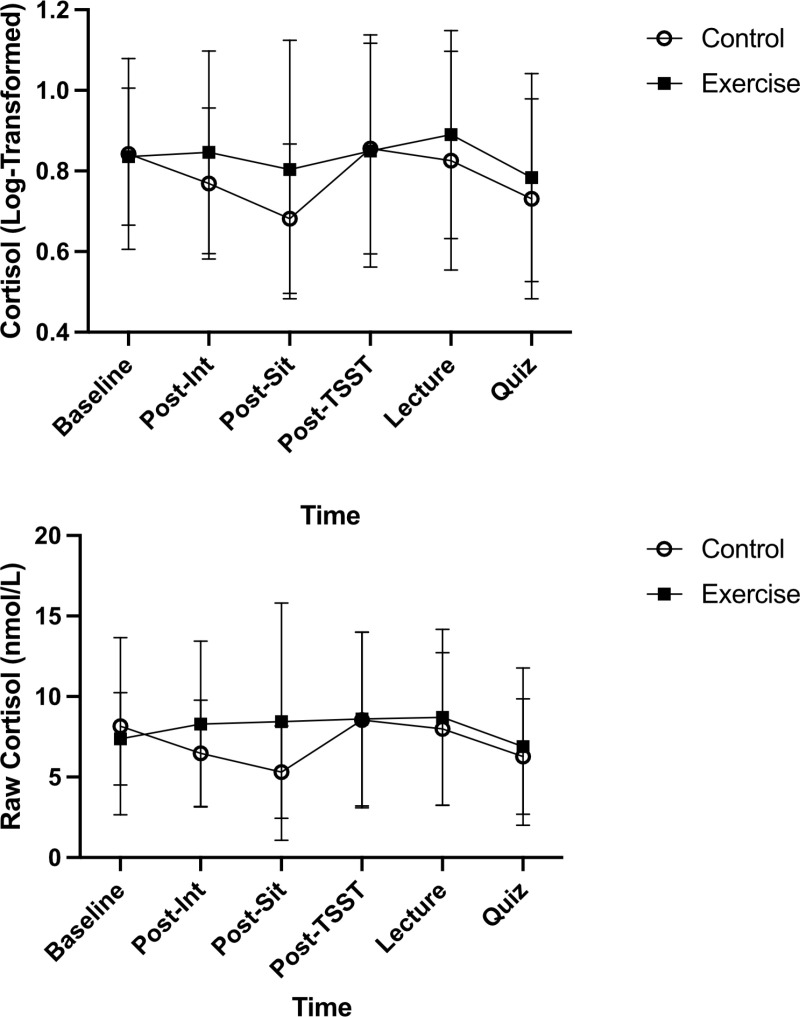
Raw and log-transformed cortisol values are depicted in the above two panels for the Control and the Exercise group. Error bars represent ± SD.

Independent t-tests for overall cortisol output, *t*(36) = 0.589, *p* = 0.560, *d* = 0.191, AUCi, *t*(36) = 1.425, *p* = 0.163, *d* = 0.463, and reactivity, *t*(36) = 1.120, *p* = 0.270, *d* = 0.364 indicated no significant differences between Control and Exercise groups. See Fig 4 for log-transformed and raw cortisol values.

#### Mind wandering probes.

Mann-Whitney U tests indicated a significant difference between the Control and Exercise group at the first checkpoint, such that there was greater mind wandering in the Control than Exercise group(U = 117.5, *p* = 0.019, *r* = 0.413) and no significant differences at the second and third checkpoints respectively (U = 138.5, *p* = 0.096, *r* = 0.308; U = 185.5, *p* = 0.698, *r* = 0.073).

#### Mind wandering minutes.

**Fig 5:**
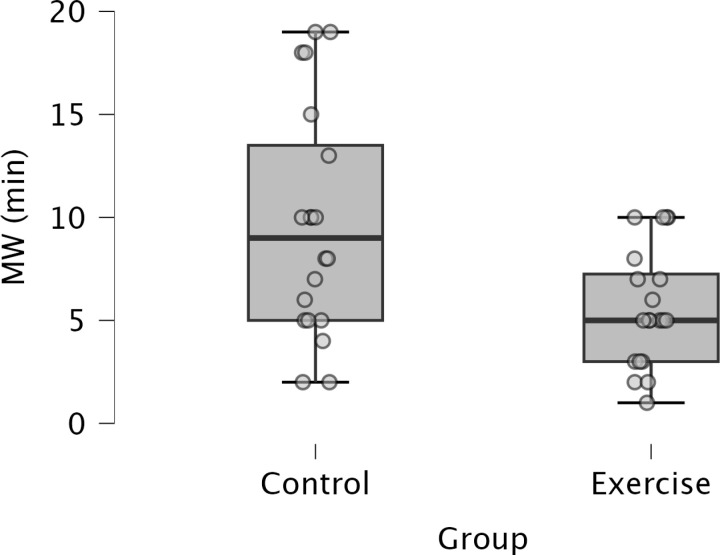
Boxplots represent the number of self-reported mind wandering minutes in the Control versus Exercise groups.

An Independent t-test indicated a significant difference in mind wandering minutes between the Control and Exercise group, such that there were greater mind wandering minutes in the Control than Exercise group, t(28.375) = 2.916, *p* = 0.007, *d* = 0.922. See Fig 5.

#### Rumination.

A Mann-Whitney U test indicated no significant difference between the Control and Exercise group in rumination scores(U = 187, *p* = 0.738, *r* = 0.065).

#### Lecture comprehension.

**Fig 6:**
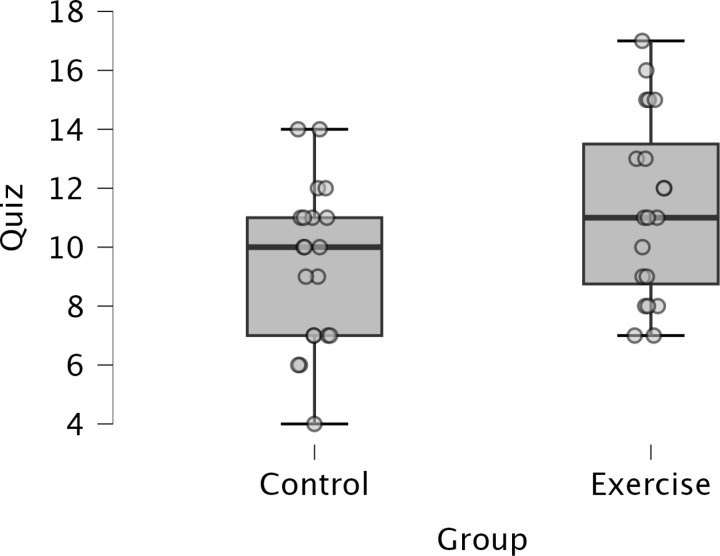
Boxplots represent the lecture comprehension score out of 20 total points in the Control versus Exercise groups.

An Independent t-test indicated a significant difference in lecture comprehension scores between the Control and Exercise group, such that the Control group had lower performance than the Exercise group, t(38) = 2.101, *p* = 0.042, *d* = 0.664. See Fig 6.

### Stress, mind wandering, and lecture comprehension associations

#### Stress measures.

When examining correlations between different stress measures in response to the TSST, Pearson’s correlations (Table 3) indicated that only HR during the TSST (Speech) was significantly positively correlated with STAI-S scores immediately post TSST (*r* = 0.320) and post lecture (*r* = 0.324).

#### Stress measures and mind wandering.

Pearson’s correlations (Table 3) indicated that STAI-S scores post lecture (*r* = 0.450) and post quiz (*r* = 0.392) were significantly positively correlated with mind wandering minutes. Spearman’s correlations (Table 2) indicated that STAI-S scores post lecture (*r* = 0.418, 0.404, 0.347) were significantly positively correlated with all three mind wandering checkpoints, while STAI-S scores post quiz (*r* = 0.362) were only significantly positively correlated with the first mind wandering checkpoint.

**Table 2:** Spearman’s correlations of probe-caught mind wandering, state anxiety, rumination, and quiz scores.

Variable		MW1	MW2	MW3	STAI_4	STAI_5	STAI_6	Quiz	Rumination
MW1	Spearman’s Rho	–							
	p-value	–							
MW2	Spearman’s Rho	0.633***	–						
	p-value	<.001	–						
MW3	Spearman’s Rho	0.496**	0.486*	–					
	p-value	0.001	0.001	–					
STAI_4	Spearman’s Rho	0.184	0.120	0.013	–				
	p-value	0.255	0.460	0.935	–				
STAI_5	Spearman’s Rho	0.418**	0.404**	0.347*	0.751***	–			
	p-value	0.007	0.010	0.028	<.001	–			
STAI_6	Spearman’s Rho	0.362*	0.245	0.207	0.685***	0.828***	–		
	p-value	0.022	0.127	0.201	<.001	<.001	–		
Quiz	Spearman’s Rho	−0.332*	−0.421**	−0.226	0.059	−0.026	0.028	–	
	p-value	0.037	0.007	0.161	0.719	0.872	0.866	–	
Rumination	Spearman’s Rho	0.337*	0.129	0.130	0.404**	0.549***	0.443**	−0.038	–
	p-value	0.033	0.428	0.425	0.010	<.001	0.004	0.817	–

*p < .05, **p < .01, ***p < 0.001.

MW1: First mind wandering probe, MW2: Second mind wandering probe, MW3: Third mind wandering probe, STAI_4: State anxiety post TSST, STAI_5: State Anxiety post lecture, STAI6_State Anxiety post Quiz, Quiz: Quiz score, Rumination: Rumination score.

#### Stress measures and lecture comprehension.

Pearson’s correlations indicated there were no significant associations between stress measures and lecture comprehension (Table 3).

**Table 3:** Pearson’s correlations of physiological variables, psychological variables, mind wandering, and lecture comprehension.

Variable		TSST Speech	TSST Math	PostTSST HR	TSST SBP	TSST DBP	STAI 4	STAI 5	STAI 6	Reactivity	AUCg	AUCi	MW (min)	Quiz
TSST Speech	Pearson’s r	–												
	p-value	–												
TSSTMath	Pearson’s r	0.607***	–											
	p-value	<.001	–											
PostTSST HR	Pearson’s r	0.669***	0.800***	–										
	p-value	<.001	<.001	–										
TSSTSBP	Pearson’s r	−0.172	0.148	0.083	–									
	p-value	0.301	0.374	0.620	–									
TSSTDBP	Pearson’s r	−0.191	0.202	0.153	0.508**	–								
	p-value	0.251	0.225	0.359	<.001	–								
STAI_4	Pearson’s r	0.293	0.084	−0.019	−0.122	−0.171	–							
	p-value	0.074	0.615	0.911	0.467	0.303	–							
STAI_5	Pearson’s r	0.292	0.219	0.088	5.48E-4	−0.148	0.722***	–						
	p-value	0.075	0.187	0.599	0.997	0.374	<.001	–						
STAI 6	Pearson’s r	0.269	0.186	0.112	−0.074	−0.156	0.687***	0.855***	–					
	p-value	0.102	0.264	0.505	0.657	0.350	<.001	<.001	–					
Reactivity	Pearson’s r	0.248	0.025	−0.025	0.153	−0.201	0.217	0.184	0.022	–				
	p-value	0.134	0.881	0.881	0.360	0.226	0.190	0.269	0.896	–				
AUCg	Pearson’s r	0.248	0.182	0.146	0.183	0.008	0.106	0.008	−0.153	0.095	–			
	p-value	0.133	0.275	0.383	0.272	0.960	0.528	0.962	0.361	0.569	–			
AUCi	Pearson’s r	0.171	−0.107	−0.081	0.212	−0.048	0.095	0.109	0.003	0.571***	0.200	–		
	p-value	0.304	0.521	0.915	0.200	0.775	0.570	0.515	0.985	<.001	0.229	–		
MW (min)	Pearson’s r	−0.091	−0.167	−0.178	0.111	−0.231	0.168	0.423**	0.355*	0.221	−0.077	0.364*	–	
	p-value	0.587	0.317	0.285	0.508	0.162	0.314	0.008	0.029	0.183	0.647	0.024	–	
Quiz	Pearson’s r	−0.128	−0.020	0.043	0.126	0.025	0.020	−0.039	−0.036	−0.047	−0.006	−0.401*	−0.423**	–
	p-value	0.443	0.905	0.799	0.452	0.882	0.904	0.817	0.831	0.779	0.973	0.013	0.008	–

*p < .05, **p < .01, ***p < 0.001.

TSSTSpeech: Speech portion of the TSST, TSSTMath: Math portion of the TSST, PostTSSTHR: Heart Rate immediately after the TSST, TSSTSBP: Systolic Blood Pressure during TSST, TSSTDBP: Diastolic Blood Pressure during TSST, STAI_4: State anxiety post TSST, STAI_5: State anxiety post lecture, STAI_6: State anxiety post quiz, Reactivity: Salivary cortisol reactivity, AUCg: Area Under the Curve with respect to ground, AUCi: Area Under the Curve with respect to increase, MW (min): Mind wandering minutes during lecture, Quiz: Quiz score.

#### Mind wandering and lecture comprehension.

Pearson’s correlation also (Table 3) indicated a significant negative correlation between mind wandering minutes and lecture comprehension (*r* = −0.417). Spearman’s correlations (Table 2) indicated significant negative correlations between mind wandering at the first (*r =* −0.332) and second checkpoints (*r =* −0.421) and lecture comprehension. See S1 Table. Correlation Tables for within-group correlations.

## Discussion

The present study examined the effects of a single 30-minute bout of high intensity aerobic exercise on acute stress responses and the downstream effects on mind wandering and lecture comprehension in young adults. Acute stress responses were not attenuated by a single bout of aerobic exercise, however individuals in the exercise group endorsed less mind wandering and higher lecture comprehension scores than those in the Control group. Notably, state anxiety scores were positively associated with mind wandering metrics and mind wandering metrics were negatively associated with lecture comprehension. Beyond these overall findings, several specific findings will be further explored.

### A single bout of exercise did not buffer acute stress responses

Contrary to our hypothesis and prior work, we did not find evidence for a reliable reduction in blood pressure and cortisol responses following a single, 30-minute bout of high intensity aerobic exercise. According to the meta-analyses by Hamer et al. (2006) and Mariano et al. (2022), statistically significant reductions to SBP and DBP have been reported following acute exercise. With respect to the magnitude of reductions for instance, Hamer et al. (2006) reported the following reductions of 3.7 ± 3.9 mmHg and 3.0 ± 2.7 mmHg for SBP and DBP respectively. In our investigation, when comparing the SBP immediately following the TSST, the Exercise group SBP is approximately 4 mmHg lower than the Control group, however, due to high variability, this difference is not statistically significant [15,16]. Importantly, in both meta-analyses, heterogeneity of findings is present as there were 5/15 [15] and 12/30 [16] studies that reported no statistically significant reductions to blood pressure measures as a result of acute exercise. The variability in findings may be due to heterogeneity in the exercise characteristics (e.g., intensity, duration, modality), acute stressor paradigm (e.g., TSST, CPT), the time delay between the exercise cessation and stressor administration, and participant characteristics [15,16,18]. For instance, in our study we utilized the TSST as the stressor, which comprises of 5-min speech and 5-min mental arithmetic as well as elements of social evaluation. In the Mariano review [16], only one study (which was not included in the meta-analysis but examined qualitatively) used the TSST. The studies included in the meta-analysis used *elements* of the TSST, such as the speech or mental arithmetic either on their own or in combination with physical stressors such as the CPT, which may explain, in part, the divergence in our findings.

With respect to cortisol, we did not detect attenuated AUCg, AUCi, or reactivity in the exercise group in comparison to the Control group. Our findings contrast prior studies examining cortisol dynamics in relation to acute exercise prior to the TSST. For instance, work by Wood et al. (2018) [46] reported lower AUCg responses in females who participated in a 30-min, moderate intensity, outdoor group walk prior to the TSST for Groups (TSST-G) in comparison to females who engaged in a non-walking group. In another investigation by Caplin and colleagues (2021) [21], the authors found an intensity-dependent effect, such that following high intensity exercise (70% HRR) for 30 minutes, salivary cortisol responses were dampened compared to lower exercise intensities (i.e., moderate and light) to the TSST. Further, they highlighted that the exercise-induced cortisol responses were inversely proportional to cortisol responses induced by the TSST. When comparing our protocol to Wood et al. (2018), they utilized walking groups, which took place in an outdoor setting, followed by the TSST-G. It is plausible that the combination of exposure to nature, which has been documented to reduce cortisol levels [47] as well as the potential protective social effects of being in a group [48] may have contributed to the buffering effect. With respect to the Caplin study [21], the authors utilized a 45-minute period between exercise cessation and TSST administration, while we employed a 30-minute period, as we were interested in utilizing a rest period found in studies examining other stress reactivity measures (e.g., SBP, DBP, state anxiety) in conjunction to cortisol. Further, they conducted a cardiopulmonary exercise treadmill test at maximal capacity to determine HR max for the calculation of HRR, while we utilized age-predicted HR max. These protocol variations may have led to differing salivary cortisol results. Of note, there also has been work documenting that 30-min of moderate-to-high aerobic exercise prior to the TSST-G did not buffer peak salivary cortisol levels [49]. Within the broader context of cortisol dynamics, although there are several studies that support the cross-stressor adaptation [17], which postulates that exercise at a sufficient intensity and duration results in adaption of the HPA axis and cortisol output, it still remains an area of further research to determine the specific parameters that dampen the HPA axis response in relation to an acute stressor such as: the time between exercise cessation and stressor onset, cardiorespiratory fitness, and the exercise environment (e.g., laboratory setting versus outdoor).

As for HR and state anxiety, there were no statistically significant differences between the exercise and Control group, which has been reported in prior acute exercise and stress investigations [18,50–52]. HR reactivity findings have been mixed in part due to measurement differences between studies. For instance, measurement differences include when HR was assessed in relation to the cessation of exercise and stressor onset, alongside the exercise intensity utilized [53]. As for acute exercise not buffering psychological responses, this may be due to decoupling of physiological and psychological responses [54] and the notion that acute exercise may not modulate the appraisal of the stressor, which is intricately linked to subsequent psychological reactivity [55]. One alternative acute intervention that has showed promise for reducing physiological and psychological responses to stress is mindfulness [56]. Theoretical work has proposed that mindfulness may mitigate “threat” stress appraisals and physiological elevations [57,58].

### A single bout of exercise promoted on-task behaviour and lecture comprehension following an acute stressor

Prior work has demonstrated acute stress increases mind wandering [13,59,60]. Notably, we have replicated our prior findings in that acute psychosocial stressors seem to exert their greatest impacts on mind wandering early in the lecture and this may be due to the proximity between stressor cessation and lecture onset. In the current study, individuals in the Control group endorsed greater mind wandering during the first lecture checkpoint than individuals in the exercise group. As for mind wandering minutes, which provide an overall subjective assessment of mind wandering behaviour, the Control group also reported greater mind wandering minutes than the exercise group (*d* = 0.922). Taken together, a single bout of exercise may play a role in promoting on-task behaviour following an acute stressor. This finding is in line with work by Fenesi et al. (2018) [40] who employed three, five-minute exercise breaks during a 50-minute video lecture and found exercise breaks promoted on-task behaviour throughout the lecture in comparison to non-exercise and no-break groups. Importantly, as we did not find support for acute exercise “buffering” stress responses, it is plausible that acute exercise boosted cognitive and executive functions, which promoted on-task behaviour, and this has been demonstrated extensively [61,62].

As the majority of studies examining acute stress and cognitive function thus far have utilized standardized, laboratory-based assessments [9,10,43] there is a limited understanding of how these findings translate to real learning environments. Although we were unable to show that multidimensional measures of acute stress relate to lecture comprehension, we do provide evidence that lecture comprehension was greater for those in the Exercise condition compared to their Control counterpart. This finding is line with meta-analysis research that shows structured classroom-based PA in the form of physically active lessons, curriculum-focused active breaks, or active breaks can increase youth academic and learning outcomes [63–65]. Overall, our findings suggest that following acute stress, exercise positively affects on-task behaviour.

### State anxiety, mind wandering, and lecture comprehension

State anxiety scores were significantly positively associated with mind wandering metrics. In other words, higher state anxiety was associated with greater probe-caught and self-reported mind wandering. These findings highlight that psychological responses to an acute stressor may be more intricately linked to the ability to stay on task, rather than physiological responses. Theoretical investigations have posited that trait anxiety reduces working memory (WM) capacity via “interference between anxiety and task related processes” [66,67]. More specifically, anxious thoughts may reduce WM capacity and reduce inhibition of task-irrelevant information [67]. The above theoretical framework has also been assessed experimentally via modulating *state anxiety* and subsequently assessing WM. For instance, Lapointe et al., 2013 [68] has documented state anxiety was associated with reduced WM capacity and inability to inhibit distracters. More recently, an investigation by Ward and colleagues (2020) induced elevations in state anxiety via brief ankle shocks to participants undergoing a visual WM task that also included distracters, but the authors only documented reductions to WM capacity [69]. Altogether, theoretical and behavioural studies provide evidence that state anxiety elevations alter cognitive processes essential to staying on task.

Consistent with a large body of work, we found mind wandering was negatively associated with lecture comprehension [34,40,70,71]. This is unsurprising as there is theoretical and behavioural evidence suggesting individuals have difficulties retaining and recalling information they were not attending to [72]. Interestingly there were no significant associations between state anxiety and lecture comprehension, which contrasts findings in the previous study [13]. This may be due, in part, to the overall scores on the quiz in this investigation being lower and less variable than scores on the prior quiz resulting in range restriction. It may also potentially suggest that it is through disruptions to mind wandering that lecture comprehension is affected, rather than state anxiety directly affecting lecture comprehension, although more work is needed to empirically assess this tenet.

### Limitations and future directions

Study strengths include the randomized control experimental design, multidimensional assessment of acute stress responses, measurement of mind wandering via probe-caught and self-report metrics, and use of a “real-life” video lecture and comprehension assessment. Limitations include the determination of exercise intensity, as we used age-predicted HRmax in our determination of HRR and this measure has been shown to demonstrate inter-participant variability [73] in comparison to measures such as V̇O_2_ max. Another limitation due to the current design, is that we cannot delineate how these findings may translate to longer lecture durations and delayed comprehension of material. Given the above, future studies should examine how various exercise characteristics (e.g., intensity, modality) may interact with acute stressors, the duration of these effects, and whether combining exercise with other interventions such as arousal reappraisal [51] and mindfulness [56] may reduce stress responses and bolster learning processes.

## Conclusion

In summary, we provide evidence that although acute exercise did not buffer stress responses induced via the TSST, individuals who engaged in acute exercise endorsed less mind wandering and improved lecture comprehension scores. Furthermore, state anxiety was positively associated with mind wandering, and mind wandering was associated with reduced lecture comprehension. Taken together, developing interventions to reduce state anxiety and mind wandering may promote optimal attention and lecture comprehension in young adults.

## Supporting information

S1 Table Within-groups correlation tables. (DOCX)

S1 Data Study3 data PLOS One. (CSV)

S2 Data Study3 data PLOS One reviewer. (CSV)
