# Supplementary material for: The effects of a single bout of high intensity exercise on stress reactivity, mind wandering, and lecture comprehension in young adults
Source: PLoS One. 2025 Jan 31;20(1):e0318222. doi: 10.1371/journal.pone.0318222 (PMC11785333; doi:10.1371/journal.pone.0318222)
Supplement: S1 Table — (DOCX) [file pone.0318222.s001.docx]

**S1. Within-Groups Correlation Tables**

**
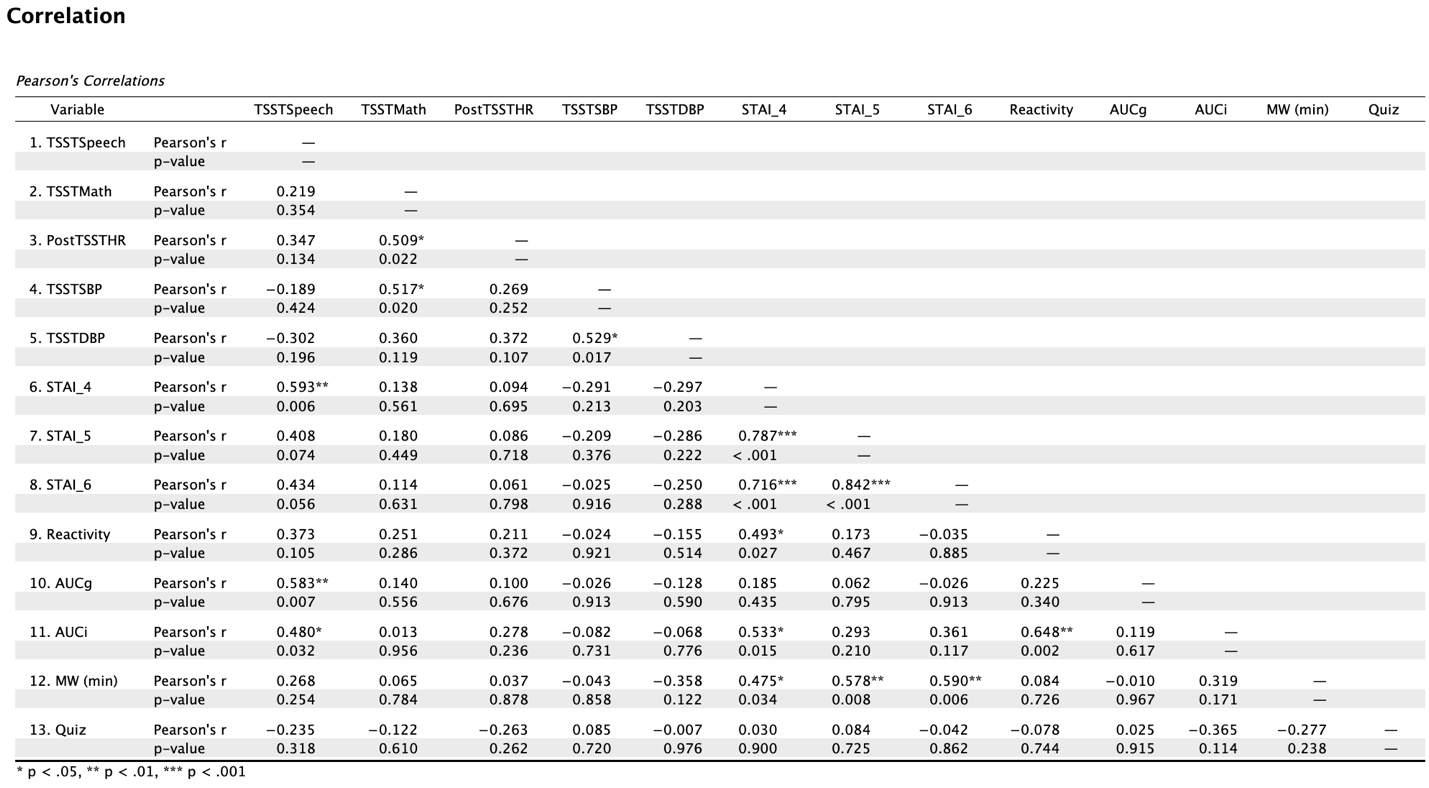
**

Table 1. Within-groups correlations (Control).


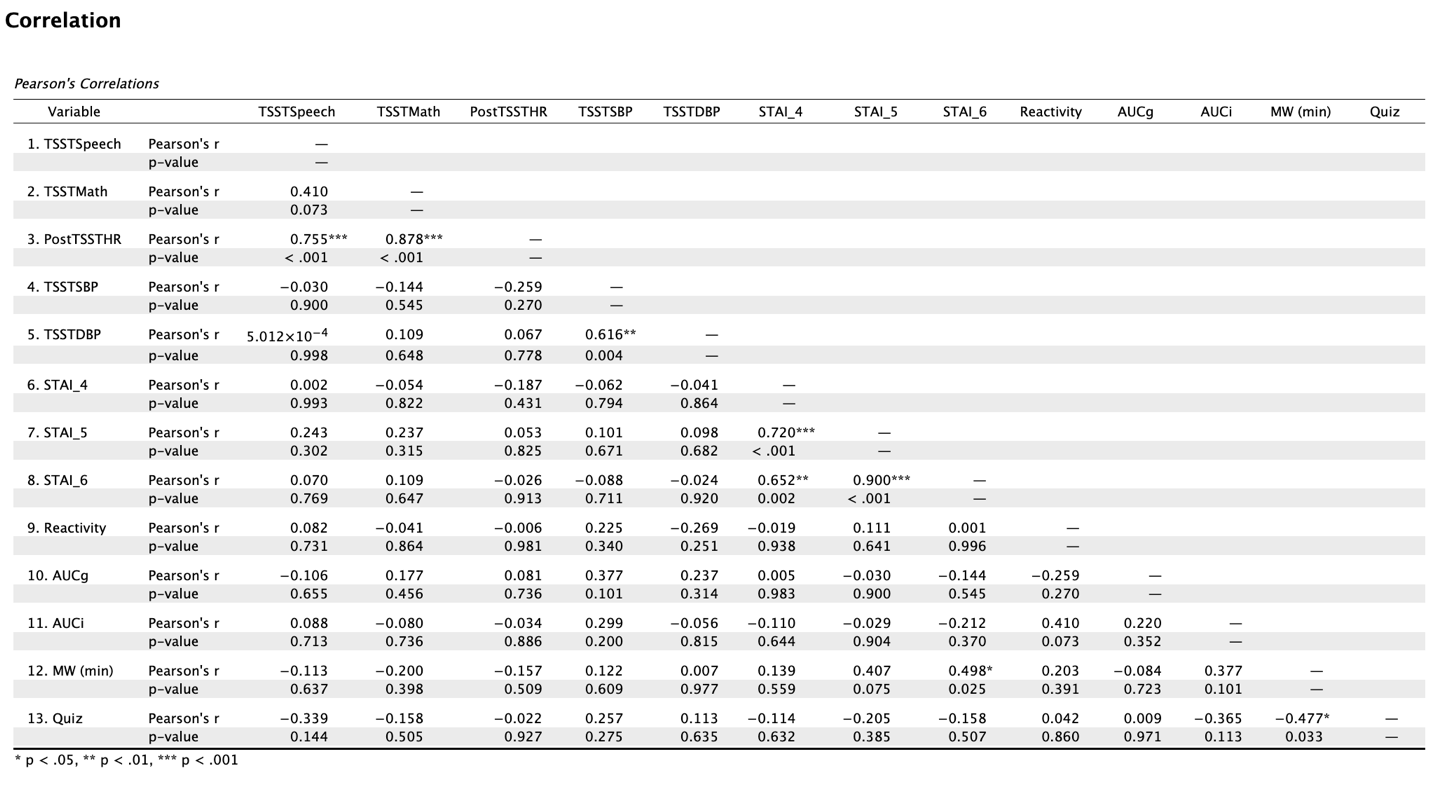


Table 2. Within-groups correlations (Exercise).
